# Chlorido(1-cyclo­pentyl­idene-4-ethyl­thio­semicarbazidato-κ^2^ *N* ^1^,*S*)diphenyl­tin(IV)

**DOI:** 10.1107/S1600536809047400

**Published:** 2009-11-21

**Authors:** Ramaiyer Venkatraman, Lungile Sitole, Frank R. Fronczek

**Affiliations:** a Department of Chemistry and Biochemistry, Jackson State University, Jackson, MS 39217, USA; b Department of Chemistry, Louisiana State University, Baton Rouge, LA 70803, USA

## Abstract

The Sn atom in the title compound, [Sn(C_6_H_5_)_2_(C_8_H_14_N_3_S)Cl], is penta­coordinated with a trigonal-bipyramidal coordination geometry. The 1-cyclo­pentyl­idene-4-ethyl­thio­semicarbazidate (cpetsc) ligand coordinates through the S atom and the N atom bonds to the cyclo­pentyl group, forming a five-membered ring with the Sn center. The chloride ligand and the coordinated N atom are in axial positions. In the crystal structure, inter­molecular N—H⋯Cl hydrogen bonds form chains along [101].

## Related literature

For the biological activity of thio­semicarbazones, see: Dogmak *et al.* (1946 ▶); Klaymann *et al.* (1979 ▶); Logan *et al.* (1975 ▶); Liberta & West (1992 ▶). For their structural characteristics, see: Livingstone (1965 ▶); Akbar & Livingstone (1974 ▶); Campbell (1975 ▶); Padhey & Kauffman (1985 ▶); Haidue & Silverstru (1990 ▶); Huheey *et al.* (1993 ▶); West *et al.* (1990 ▶, 1993 ▶); Lobana *et al.* (2009 ▶). For the anti­tumor activity of organotin(IV) complexes, see: Nath *et al.* (2001 ▶); Pellerito & Nagy (2002 ▶). For related structures, see: Swesi *et al.* (2005 ▶, 2006 ▶); Valente *et al.* (1998 ▶); Huheey *et al.* (1993 ▶); Venkatraman *et al.* (1999 ▶); Pal *et al.* (2002 ▶); Teoh *et al.* (1999 ▶).
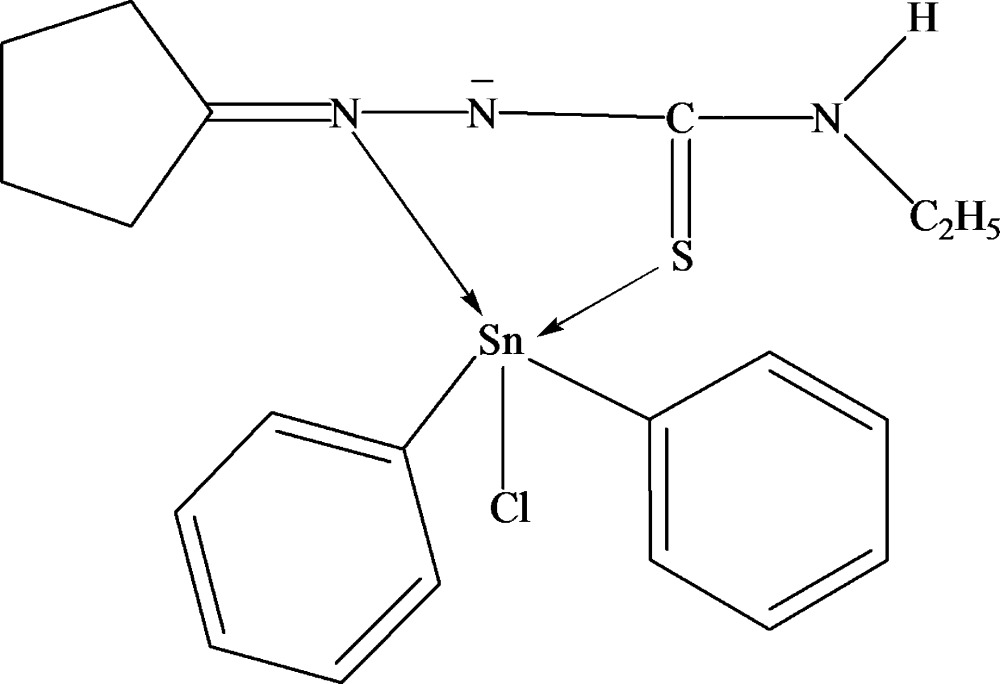


## Experimental

### 

#### Crystal data

[Sn(C_6_H_5_)_2_(C_8_H_14_N_3_S)Cl]
*M* *_r_* = 492.62
Monoclinic, 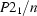

*a* = 8.9031 (9) Å
*b* = 22.951 (3) Å
*c* = 11.1381 (11) Å
β = 111.094 (4)°
*V* = 2123.4 (4) Å^3^
*Z* = 4
Mo *K*α radiation
μ = 1.44 mm^−1^
*T* = 90 K
0.27 × 0.23 × 0.17 mm

#### Data collection

Nonius KappaCCD diffractometer with an Oxford Cryosystems Cryostream cooler
Absorption correction: multi-scan (*SCALEPACK*; Otwinowski & Minor, 1997 ▶) *T*_min_ = 0.698, *T*_max_ = 0.792
33496 measured reflections
8756 independent reflections
7543 reflections with *I* > 2σ(*I*)
*R*_int_ = 0.022

#### Refinement

*R*[*F* ^2^ > 2σ(*F* ^2^)] = 0.026
*wR*(*F* ^2^) = 0.062
*S* = 1.04
8756 reflections
240 parameters
H atoms treated by a mixture of independent and constrained refinement
Δρ_max_ = 0.64 e Å^−3^
Δρ_min_ = −1.38 e Å^−3^

### 

Data collection: *COLLECT* (Nonius 2000 ▶); cell refinement: *DENZO* and *SCALEPACK* (Otwinowski & Minor, 1997 ▶); data reduction: *DENZO* and *SCALEPACK*; program(s) used to solve structure: *SIR* (Altomare *et al.*, 1999 ▶); program(s) used to refine structure: *SHELXL97* (Sheldrick, 2008 ▶); molecular graphics: *ORTEP-3 for Windows* (Farrugia, 1997 ▶); software used to prepare material for publication: *SHELXL97*.

## Supplementary Material

Crystal structure: contains datablocks I, global. DOI: 10.1107/S1600536809047400/lh2925sup1.cif

Structure factors: contains datablocks I. DOI: 10.1107/S1600536809047400/lh2925Isup2.hkl

Additional supplementary materials:  crystallographic information; 3D view; checkCIF report

## Figures and Tables

**Table 1:** Selected bond lengths (Å)

Sn1—C21	2.1331 (14)
Sn1—C31	2.1397 (14)
Sn1—N1	2.3123 (12)
Sn1—S1	2.4363 (4)
Sn1—Cl1	2.5095 (4)

**Table 2:** Hydrogen-bond geometry (Å, °)

*D*—H⋯*A*	*D*—H	H⋯*A*	*D*⋯*A*	*D*—H⋯*A*
N3—H3*N*⋯Cl1^i^	0.80 (2)	2.71 (2)	3.4731 (15)	160 (2)

Symmetry code: (i) 
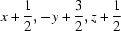
.

**Fig. 1.**
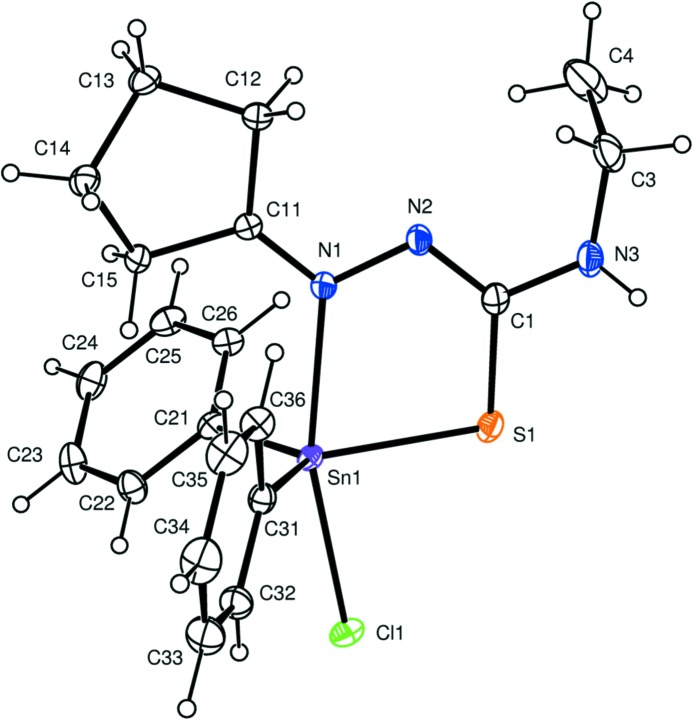
The molecular structure of the title compound with the numbering scheme and ellipsoids at the 50% level.

**Fig. 2.**
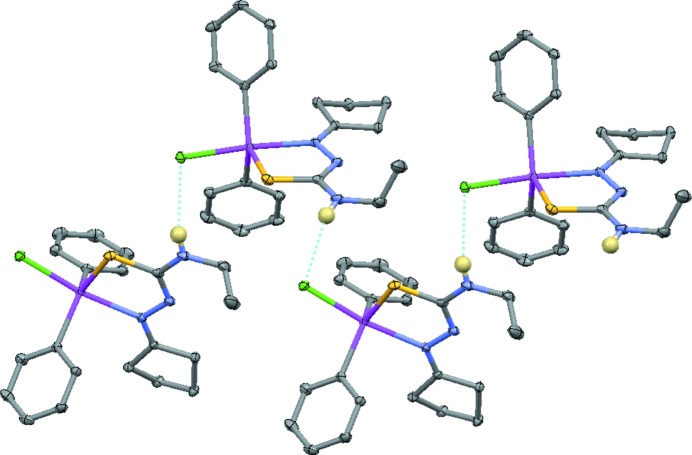
A portion of the hydrogen-bonded chain extending in along [1 0 1]. Hydrogen bonds are shown as dotted lines.

## supplementary crystallographic information

### Comment

Thiosemicarbazones can contain chemically active –N(H)C(S) or NN(H)C(S) chromophores which make them unique in their reactivity. They are also well known for biological activities (Klaymann *et al.*, 1979; Logan *et al.*, 1975) since the original discovery of their anti-tubercular activity by Dogmak *et al.* (1946). Many review articles have appeared in the literature highlighting their structural characteristics (Livingstone 1965; Akbar & Livingstone, 1974, Campbell, 1975; Padhey & Kauffman, 1985; Haidue & Silverstru, 1990; West *et al.*, 1990; 1993; Lobana *et al.*, 2009). Among the non-transition metallo-pharmaceuticals, organotin (IV) complexes have demonstrated relatively high antitumor activity (Nath *et al.*, 2001, Pellerito & Nagy, 2002). The present report describes the structure of a diiorganotin(IV) complex with N4 ethyl-substituted cyclopentanothiosemicarbazone, cpetsc.

The reaction of cpetsc with SnPh_2_Cl_2_ formed a monomeric anionic complex (see Fig. 1). The geometry of the tin(IV) center is penta-coordinated with a distorted trigonal bipyramidal (TBP) geometry. The two phenyl carbons C21 and C31 are positioned at the equatorial plane, while the azomethine nitrogen N1 and chlorine atoms, Cl1 occupy the axial positions. The bond distances involving the Sn atom are comparable to the reported values for dimethyl and diphenyl tin(IV) complexes of acetone (Swesi *et al.*, 2005, Swesi *et al.*, 2006) and diphenyl tin(IV) dichloro thiophene-2-carboxaldehyde (Teoh *et al.* 1999).

Significant lengthening of the C ═S bond and shortening of the C—N bond is observed as compared with the parent ligand (Valente *et al.*, 1998, Venkatraman *et al.*, 1999). The C—S bond distance (1.7709 Å) is relatively shorter than a single bond distance (1.81 Å) but longer than a C—S double bond (1.62 Å) distance (Huheey *et al.*, 1993). The nature of coordination exhibited by the thiosemicarbazones are mainly due to the E and *Z* configuration of the ligand and the mode of coordination depends on the steric bulk of the carbonylic carbon atom linked *trans* to the hydrazinic nitrogen. A stable five-membered ring will be formed if the carbonylic carbon carries a small group, or else a four membered ring with larger group (Pal *et al.*, 2002). In the present case, the metal is bound by the thiosemicarbazone, forming a five-membered chelate ring.

N—H···Cl intermolecular hydrogen bonds form chains along [1 0 1], as shown in Figure 2.

### Experimental

A solution of diphenyltindichloride (0.344 g, 1.0 mmol) in dry methanol (10 ml) was added slowly to a boiling solution of cyclopentano-4-ethyl-3-thiosemicarbazone (0.185 g, 1.0 mmol). in methanol (50 ml) (Valente *et al.*, 1998, Venkatraman *et al.*, 1999). The resulting mixture was refluxed for a period of 2 h and then allowed to cool to room temperature in presence of air. Colorless rods of the title complex were obtained on slow evaporation of the solvent (yield approx. 65%, mp 460–462 K) at room temperature (C_20_H_24_ClN_3_SSn, C, 47.55%, H, 5.45%, N, 8.48%, S, 6.45%). ^1^HNMR: 7.67ppm, 9.73ppm. Main IR peaks (KBr): ν_N—H_ 3400, 3305, 3080 cm^-1^, ν_C=S_ 820, 730, 705 cm^-1^.

### Refinement

Hydrogen atoms were placed in idealized positions, with C—H bond distances 0.95 - 0.99 Å, and thereafter treated as riding. Displacement parameters for H were assigned as *U*_iso_ = 1.2Ueq of the attached atom (1.5 for methyl). A torsional parameter was refined for the methyl group. The largest negative residual difference map peak is 0.74 Å from the Sn atom.

### Crystal data

**Table:**

[Sn(C_6_H_5_)_2_(C_8_H_14_N_3_S)Cl]	*F*(000) = 992
*M**_r_* = 492.62	*D*_x_ = 1.541 Mg m^−^^3^
Monoclinic, *P*2_1_/*n*	Mo *K*α radiation, λ = 0.71073 Å
Hall symbol: -P 2yn	Cell parameters from 8037 reflections
*a* = 8.9031 (9) Å	θ = 2.5–34.8°
*b* = 22.951 (3) Å	µ = 1.44 mm^−^^1^
*c* = 11.1381 (11) Å	*T* = 90 K
β = 111.094 (4)°	Fragment, colorless
*V* = 2123.4 (4) Å^3^	0.27 × 0.23 × 0.17 mm
*Z* = 4	

### Data collection

**Table:**

Nonius KappaCCD diffractometer with an Oxford Cryosystems Cryostream cooler	8756 independent reflections
Radiation source: fine-focus sealed tube	7543 reflections with *I* > 2σ(*I*)
graphite	*R*_int_ = 0.022
ω and φ scans	θ_max_ = 34.8°, θ_min_ = 3.0°
Absorption correction: multi-scan (*SCALEPACK*; Otwinowski & Minor, 1997)	*h* = −13→13
*T*_min_ = 0.698, *T*_max_ = 0.792	*k* = −35→35
33496 measured reflections	*l* = −17→16

### Refinement

**Table:**

Refinement on *F*^2^	Secondary atom site location: difference Fourier map
Least-squares matrix: full	Hydrogen site location: inferred from neighbouring sites
*R*[*F*^2^ > 2σ(*F*^2^)] = 0.026	H atoms treated by a mixture of independent and constrained refinement
*wR*(*F*^2^) = 0.062	*w* = 1/[σ^2^(*F*_o_^2^) + (0.0232*P*)^2^ + 1.4419*P*] where *P* = (*F*_o_^2^ + 2*F*_c_^2^)/3
*S* = 1.04	(Δ/σ)_max_ = 0.002
8756 reflections	Δρ_max_ = 0.64 e Å^−^^3^
240 parameters	Δρ_min_ = −1.38 e Å^−^^3^
0 restraints	Extinction correction: *SHELXL97* (Sheldrick, 2008), Fc^*^=kFc[1+0.001xFc^2^λ^3^/sin(2θ)]^-1/4^
Primary atom site location: structure-invariant direct methods	Extinction coefficient: 0.00150 (18)

### Special details

**Table:**

Geometry. All e.s.d.'s (except the e.s.d. in the dihedral angle between two l.s. planes) are estimated using the full covariance matrix. The cell e.s.d.'s are taken into account individually in the estimation of e.s.d.'s in distances, angles and torsion angles; correlations between e.s.d.'s in cell parameters are only used when they are defined by crystal symmetry. An approximate (isotropic) treatment of cell e.s.d.'s is used for estimating e.s.d.'s involving l.s. planes.
Refinement. Refinement of *F*^2^ against ALL reflections. The weighted *R*-factor *wR* and goodness of fit *S* are based on *F*^2^, conventional *R*-factors *R* are based on *F*, with *F* set to zero for negative *F*^2^. The threshold expression of *F*^2^ > σ(*F*^2^) is used only for calculating *R*-factors(gt) *etc*. and is not relevant to the choice of reflections for refinement. *R*-factors based on *F*^2^ are statistically about twice as large as those based on *F*, and *R*- factors based on ALL data will be even larger.

### Fractional atomic coordinates and isotropic or equivalent isotropic displacement parameters (Å^2^)

**Table:**

	*x*	*y*	*z*	*U*_iso_*/*U*_eq_	
Sn1	0.542305 (11)	0.626341 (4)	0.258844 (9)	0.01109 (3)	
Cl1	0.25741 (4)	0.662360 (15)	0.18735 (4)	0.01732 (7)	
S1	0.58505 (5)	0.680095 (16)	0.45705 (4)	0.01588 (7)	
N1	0.80541 (14)	0.60297 (5)	0.38227 (11)	0.0127 (2)	
N2	0.88356 (16)	0.63137 (5)	0.49842 (12)	0.0148 (2)	
N3	0.86198 (18)	0.69504 (6)	0.64811 (13)	0.0192 (2)	
H3N	0.817 (3)	0.7237 (10)	0.660 (2)	0.023*	
C1	0.79332 (18)	0.66634 (6)	0.53568 (14)	0.0147 (2)	
C3	1.0332 (2)	0.68887 (7)	0.72462 (17)	0.0249 (3)	
H3A	1.0694	0.7230	0.7822	0.030*	
H3B	1.0951	0.6882	0.6665	0.030*	
C4	1.0678 (3)	0.63345 (8)	0.8051 (2)	0.0403 (6)	
H4A	1.0173	0.6360	0.8699	0.060*	
H4B	1.1844	0.6288	0.8480	0.060*	
H4C	1.0241	0.5998	0.7491	0.060*	
C11	0.89645 (16)	0.56885 (6)	0.34655 (13)	0.0125 (2)	
C12	1.07472 (17)	0.56124 (7)	0.41598 (14)	0.0154 (2)	
H12A	1.0983	0.5465	0.5045	0.018*	
H12B	1.1324	0.5985	0.4204	0.018*	
C13	1.12338 (17)	0.51630 (7)	0.33416 (15)	0.0167 (3)	
H13A	1.1191	0.4762	0.3656	0.020*	
H13B	1.2333	0.5241	0.3355	0.020*	
C14	0.99740 (17)	0.52473 (7)	0.19811 (14)	0.0161 (3)	
H14A	1.0232	0.5591	0.1553	0.019*	
H14B	0.9901	0.4898	0.1441	0.019*	
C15	0.84068 (17)	0.53416 (6)	0.22362 (13)	0.0140 (2)	
H15A	0.7610	0.5562	0.1527	0.017*	
H15B	0.7927	0.4966	0.2348	0.017*	
C21	0.47816 (16)	0.53643 (6)	0.24833 (13)	0.0128 (2)	
C22	0.36518 (19)	0.51214 (7)	0.13708 (15)	0.0179 (3)	
H22	0.3148	0.5359	0.0636	0.021*	
C23	0.3266 (2)	0.45318 (7)	0.13406 (16)	0.0223 (3)	
H23	0.2500	0.4368	0.0584	0.027*	
C24	0.3997 (2)	0.41813 (7)	0.24138 (17)	0.0215 (3)	
H24	0.3732	0.3779	0.2388	0.026*	
C25	0.51138 (19)	0.44199 (7)	0.35228 (16)	0.0188 (3)	
H25	0.5609	0.4182	0.4257	0.023*	
C26	0.55061 (17)	0.50098 (6)	0.35548 (14)	0.0152 (2)	
H26	0.6273	0.5172	0.4313	0.018*	
C31	0.60608 (17)	0.67142 (6)	0.11590 (14)	0.0140 (2)	
C32	0.4852 (2)	0.69413 (7)	0.00748 (15)	0.0183 (3)	
H32	0.3754	0.6884	−0.0034	0.022*	
C33	0.5245 (2)	0.72517 (8)	−0.08492 (16)	0.0243 (3)	
H33	0.4414	0.7401	−0.1586	0.029*	
C34	0.6850 (2)	0.73426 (8)	−0.06946 (17)	0.0257 (3)	
H34	0.7114	0.7559	−0.1319	0.031*	
C35	0.8061 (2)	0.71168 (7)	0.03722 (17)	0.0235 (3)	
H35	0.9157	0.7175	0.0475	0.028*	
C36	0.76722 (18)	0.68049 (6)	0.12944 (15)	0.0171 (3)	
H36	0.8509	0.6652	0.2024	0.021*	

### Atomic displacement parameters (Å^2^)

**Table:**

	*U*^11^	*U*^22^	*U*^33^	*U*^12^	*U*^13^	*U*^23^
Sn1	0.01215 (4)	0.00957 (4)	0.01273 (5)	0.00074 (3)	0.00592 (3)	0.00121 (3)
Cl1	0.01357 (14)	0.01546 (15)	0.02368 (16)	0.00282 (11)	0.00761 (12)	0.00180 (12)
S1	0.01974 (16)	0.01448 (15)	0.01573 (15)	0.00316 (12)	0.00920 (13)	−0.00064 (12)
N1	0.0134 (5)	0.0116 (5)	0.0126 (5)	0.0001 (4)	0.0042 (4)	−0.0010 (4)
N2	0.0179 (5)	0.0123 (5)	0.0132 (5)	0.0000 (4)	0.0043 (4)	−0.0020 (4)
N3	0.0266 (7)	0.0136 (5)	0.0163 (6)	0.0001 (5)	0.0065 (5)	−0.0036 (4)
C1	0.0210 (6)	0.0101 (5)	0.0132 (6)	−0.0003 (5)	0.0065 (5)	0.0015 (4)
C3	0.0310 (9)	0.0161 (7)	0.0201 (7)	−0.0033 (6)	0.0003 (6)	−0.0042 (6)
C4	0.0507 (13)	0.0213 (8)	0.0283 (9)	−0.0043 (8)	−0.0106 (9)	0.0016 (7)
C11	0.0122 (5)	0.0121 (5)	0.0134 (6)	0.0001 (4)	0.0049 (4)	0.0007 (4)
C12	0.0120 (6)	0.0172 (6)	0.0158 (6)	0.0005 (5)	0.0038 (5)	−0.0010 (5)
C13	0.0131 (6)	0.0202 (7)	0.0182 (6)	0.0029 (5)	0.0073 (5)	−0.0001 (5)
C14	0.0157 (6)	0.0189 (6)	0.0159 (6)	0.0021 (5)	0.0084 (5)	0.0003 (5)
C15	0.0137 (6)	0.0150 (6)	0.0137 (6)	0.0009 (5)	0.0056 (5)	−0.0020 (5)
C21	0.0131 (6)	0.0124 (5)	0.0154 (6)	0.0006 (4)	0.0080 (5)	0.0011 (5)
C22	0.0222 (7)	0.0173 (6)	0.0140 (6)	−0.0023 (5)	0.0065 (5)	0.0006 (5)
C23	0.0302 (8)	0.0199 (7)	0.0187 (7)	−0.0074 (6)	0.0110 (6)	−0.0058 (6)
C24	0.0285 (8)	0.0121 (6)	0.0283 (8)	−0.0029 (5)	0.0157 (6)	−0.0023 (6)
C25	0.0190 (7)	0.0139 (6)	0.0248 (7)	0.0017 (5)	0.0094 (6)	0.0057 (5)
C26	0.0145 (6)	0.0144 (6)	0.0171 (6)	−0.0002 (5)	0.0063 (5)	0.0026 (5)
C31	0.0169 (6)	0.0117 (5)	0.0143 (6)	−0.0002 (5)	0.0070 (5)	0.0000 (5)
C32	0.0214 (7)	0.0174 (6)	0.0153 (6)	0.0010 (5)	0.0056 (5)	0.0017 (5)
C33	0.0334 (9)	0.0227 (7)	0.0167 (7)	0.0036 (6)	0.0090 (6)	0.0058 (6)
C34	0.0388 (10)	0.0217 (8)	0.0233 (8)	−0.0003 (7)	0.0191 (7)	0.0065 (6)
C35	0.0273 (8)	0.0206 (7)	0.0289 (8)	−0.0028 (6)	0.0178 (7)	0.0037 (6)
C36	0.0182 (6)	0.0149 (6)	0.0196 (7)	−0.0015 (5)	0.0085 (5)	0.0017 (5)

### Geometric parameters (Å, °)

**Table:**

Sn1—C21	2.1331 (14)	C14—H14A	0.9900
Sn1—C31	2.1397 (14)	C14—H14B	0.9900
Sn1—N1	2.3123 (12)	C15—H15A	0.9900
Sn1—S1	2.4363 (4)	C15—H15B	0.9900
Sn1—Cl1	2.5095 (4)	C21—C26	1.396 (2)
S1—C1	1.7709 (16)	C21—C22	1.400 (2)
N1—C11	1.2887 (18)	C22—C23	1.394 (2)
N1—N2	1.3909 (17)	C22—H22	0.9500
N2—C1	1.3047 (19)	C23—C24	1.393 (2)
N3—C1	1.3508 (19)	C23—H23	0.9500
N3—C3	1.461 (2)	C24—C25	1.389 (2)
N3—H3N	0.80 (2)	C24—H24	0.9500
C3—C4	1.522 (3)	C25—C26	1.395 (2)
C3—H3A	0.9900	C25—H25	0.9500
C3—H3B	0.9900	C26—H26	0.9500
C4—H4A	0.9800	C31—C32	1.397 (2)
C4—H4B	0.9800	C31—C36	1.403 (2)
C4—H4C	0.9800	C32—C33	1.396 (2)
C11—C12	1.5047 (19)	C32—H32	0.9500
C11—C15	1.505 (2)	C33—C34	1.392 (3)
C12—C13	1.538 (2)	C33—H33	0.9500
C12—H12A	0.9900	C34—C35	1.386 (3)
C12—H12B	0.9900	C34—H34	0.9500
C13—C14	1.539 (2)	C35—C36	1.394 (2)
C13—H13A	0.9900	C35—H35	0.9500
C13—H13B	0.9900	C36—H36	0.9500
C14—C15	1.536 (2)		
			
C21—Sn1—C31	124.36 (5)	C15—C14—C13	102.96 (12)
C21—Sn1—N1	90.12 (5)	C15—C14—H14A	111.2
C31—Sn1—N1	94.09 (5)	C13—C14—H14A	111.2
C21—Sn1—S1	119.41 (4)	C15—C14—H14B	111.2
C31—Sn1—S1	115.63 (4)	C13—C14—H14B	111.2
N1—Sn1—S1	77.54 (3)	H14A—C14—H14B	109.1
C21—Sn1—Cl1	94.66 (4)	C11—C15—C14	102.62 (11)
C31—Sn1—Cl1	96.50 (4)	C11—C15—H15A	111.2
N1—Sn1—Cl1	163.15 (3)	C14—C15—H15A	111.2
S1—Sn1—Cl1	86.050 (13)	C11—C15—H15B	111.2
C1—S1—Sn1	98.75 (5)	C14—C15—H15B	111.2
C11—N1—N2	114.34 (12)	H15A—C15—H15B	109.2
C11—N1—Sn1	124.93 (9)	C26—C21—C22	119.24 (13)
N2—N1—Sn1	120.33 (9)	C26—C21—Sn1	118.99 (10)
C1—N2—N1	115.15 (12)	C22—C21—Sn1	121.77 (10)
C1—N3—C3	121.50 (14)	C23—C22—C21	120.02 (14)
C1—N3—H3N	117.8 (16)	C23—C22—H22	120.0
C3—N3—H3N	117.2 (16)	C21—C22—H22	120.0
N2—C1—N3	118.03 (14)	C24—C23—C22	120.34 (15)
N2—C1—S1	127.40 (11)	C24—C23—H23	119.8
N3—C1—S1	114.56 (11)	C22—C23—H23	119.8
N3—C3—C4	111.85 (16)	C25—C24—C23	119.94 (14)
N3—C3—H3A	109.2	C25—C24—H24	120.0
C4—C3—H3A	109.2	C23—C24—H24	120.0
N3—C3—H3B	109.2	C24—C25—C26	119.85 (14)
C4—C3—H3B	109.2	C24—C25—H25	120.1
H3A—C3—H3B	107.9	C26—C25—H25	120.1
C3—C4—H4A	109.5	C25—C26—C21	120.61 (14)
C3—C4—H4B	109.5	C25—C26—H26	119.7
H4A—C4—H4B	109.5	C21—C26—H26	119.7
C3—C4—H4C	109.5	C32—C31—C36	118.50 (14)
H4A—C4—H4C	109.5	C32—C31—Sn1	119.69 (11)
H4B—C4—H4C	109.5	C36—C31—Sn1	121.78 (11)
N1—C11—C12	125.18 (13)	C33—C32—C31	120.55 (15)
N1—C11—C15	124.34 (12)	C33—C32—H32	119.7
C12—C11—C15	110.35 (12)	C31—C32—H32	119.7
C11—C12—C13	104.07 (11)	C34—C33—C32	120.25 (16)
C11—C12—H12A	110.9	C34—C33—H33	119.9
C13—C12—H12A	110.9	C32—C33—H33	119.9
C11—C12—H12B	110.9	C35—C34—C33	119.81 (15)
C13—C12—H12B	110.9	C35—C34—H34	120.1
H12A—C12—H12B	109.0	C33—C34—H34	120.1
C12—C13—C14	103.78 (11)	C34—C35—C36	120.07 (16)
C12—C13—H13A	111.0	C34—C35—H35	120.0
C14—C13—H13A	111.0	C36—C35—H35	120.0
C12—C13—H13B	111.0	C35—C36—C31	120.82 (15)
C14—C13—H13B	111.0	C35—C36—H36	119.6
H13A—C13—H13B	109.0	C31—C36—H36	119.6
			
C21—Sn1—S1—C1	89.47 (6)	C31—Sn1—C21—C26	131.64 (11)
C31—Sn1—S1—C1	−82.09 (6)	N1—Sn1—C21—C26	36.61 (11)
N1—Sn1—S1—C1	6.48 (6)	S1—Sn1—C21—C26	−39.13 (12)
Cl1—Sn1—S1—C1	−177.41 (5)	Cl1—Sn1—C21—C26	−127.21 (11)
C21—Sn1—N1—C11	59.16 (12)	C31—Sn1—C21—C22	−48.53 (14)
C31—Sn1—N1—C11	−65.32 (12)	N1—Sn1—C21—C22	−143.57 (12)
S1—Sn1—N1—C11	179.32 (12)	S1—Sn1—C21—C22	140.69 (11)
Cl1—Sn1—N1—C11	165.84 (8)	Cl1—Sn1—C21—C22	52.61 (12)
C21—Sn1—N1—N2	−128.54 (10)	C26—C21—C22—C23	−0.2 (2)
C31—Sn1—N1—N2	106.99 (10)	Sn1—C21—C22—C23	180.00 (12)
S1—Sn1—N1—N2	−8.38 (9)	C21—C22—C23—C24	0.1 (3)
Cl1—Sn1—N1—N2	−21.85 (18)	C22—C23—C24—C25	0.2 (3)
C11—N1—N2—C1	179.64 (13)	C23—C24—C25—C26	−0.3 (2)
Sn1—N1—N2—C1	6.56 (16)	C24—C25—C26—C21	0.2 (2)
N1—N2—C1—N3	179.70 (12)	C22—C21—C26—C25	0.0 (2)
N1—N2—C1—S1	1.32 (19)	Sn1—C21—C26—C25	179.85 (11)
C3—N3—C1—N2	1.9 (2)	C21—Sn1—C31—C32	84.09 (13)
C3—N3—C1—S1	−179.53 (12)	N1—Sn1—C31—C32	177.03 (12)
Sn1—S1—C1—N2	−7.17 (14)	S1—Sn1—C31—C32	−104.83 (11)
Sn1—S1—C1—N3	174.40 (10)	Cl1—Sn1—C31—C32	−16.10 (12)
C1—N3—C3—C4	−80.7 (2)	C21—Sn1—C31—C36	−97.81 (13)
N2—N1—C11—C12	−3.6 (2)	N1—Sn1—C31—C36	−4.86 (12)
Sn1—N1—C11—C12	169.12 (10)	S1—Sn1—C31—C36	73.28 (12)
N2—N1—C11—C15	−178.95 (12)	Cl1—Sn1—C31—C36	162.00 (11)
Sn1—N1—C11—C15	−6.2 (2)	C36—C31—C32—C33	−0.1 (2)
N1—C11—C12—C13	179.96 (14)	Sn1—C31—C32—C33	178.09 (12)
C15—C11—C12—C13	−4.12 (16)	C31—C32—C33—C34	−0.5 (3)
C11—C12—C13—C14	27.08 (15)	C32—C33—C34—C35	0.9 (3)
C12—C13—C14—C15	−39.94 (14)	C33—C34—C35—C36	−0.7 (3)
N1—C11—C15—C14	155.55 (14)	C34—C35—C36—C31	0.1 (3)
C12—C11—C15—C14	−20.40 (15)	C32—C31—C36—C35	0.3 (2)
C13—C14—C15—C11	36.64 (14)	Sn1—C31—C36—C35	−177.83 (12)

### Hydrogen-bond geometry (Å, °)

**Table:**

*D*—H···*A*	*D*—H	H···*A*	*D*···*A*	*D*—H···*A*
N3—H3N···Cl1^i^	0.80 (2)	2.71 (2)	3.4731 (15)	160 (2)

Symmetry codes: (i) *x*+1/2, −*y*+3/2, *z*+1/2.
